# Marked TGF-β-regulated miRNA expression changes in both COPD and control lung fibroblasts

**DOI:** 10.1038/s41598-019-54728-4

**Published:** 2019-12-03

**Authors:** J. Ong, A. Faiz, W. Timens, M. van den Berge, M. M. Terpstra, K. Kok, A. van den Berg, J. Kluiver, C. A. Brandsma

**Affiliations:** 1 University of Groningen, University Medical Centre Groningen, Department of Pathology and Medical Biology, Groningen, The Netherlands; 2 University of Groningen, University Medical Centre Groningen, Groningen Research Institute for Asthma and COPD (GRIAC), Groningen, The Netherlands; 3 University of Groningen, University Medical Centre Groningen, Department of Pulmonary Diseases, Groningen, The Netherlands; 4 University of Technology Sydney, Respiratory Bioinformatics and Molecular Biology (RBMB) Faculty of Science, Ultimo, NSW 2007 Australia; 5 University of Groningen, University Medical Centre Groningen, Department of Genetics, Groningen, The Netherlands

**Keywords:** miRNAs, Chronic obstructive pulmonary disease

## Abstract

COPD is associated with disturbed tissue repair, possibly due to TGF-β-regulated miRNA changes in fibroblasts. Our aim was to identify TGF-β-regulated miRNAs and their differential regulation and expression in COPD compared to control fibroblasts. Small RNA sequencing was performed on TGF-β-stimulated and unstimulated lung fibroblasts from 15 COPD patients and 15 controls. Linear regression was used to identify TGF-β-regulated and COPD-associated miRNAs. Interaction analysis was performed to compare miRNAs that responded differently to TGF-β in COPD and control. Re-analysis of previously generated Ago2-IP data and Enrichr were used to identify presence and function of potential target genes in the miRNA-targetome of lung fibroblasts. In total, 46 TGF-β-regulated miRNAs were identified in COPD and 86 in control fibroblasts (FDR < 0.05). MiR-27a-5p was the most significantly upregulated miRNA. MiR-148b-3p, miR-589-5p and miR-376b-3p responded differently to TGF-β in COPD compared to control (FDR < 0.25). MiR-660-5p was significantly upregulated in COPD compared to control (FDR < 0.05). Several predicted targets of miR-27a-5p, miR-148b-3p and miR-660-5p were present in the miRNA-targetome, and were mainly involved in the regulation of gene transcription. In conclusion, altered TGF-β-induced miRNA regulation and differential expression of miR-660-5p in COPD fibroblasts, may represent one of the mechanisms underlying aberrant tissue repair and remodelling in COPD.

## Introduction

Chronic obstructive pulmonary disease (COPD) is characterized by a variable extent and combination of (small) airway disease and emphysema. It is a major and still growing cause of death worldwide^1,2^. One of the features underlying the development of COPD is aberrant tissue repair and remodelling^3^, leading to a disturbed extracellular matrix (ECM) homeostasis. In COPD patients, narrowing of the (small) airways is observed, due to airway wall fibrosis^4^. In contrast, proper tissue repair is diminished in the parenchyma contributing to the development of pulmonary emphysema^4,5^. Malfunction of lung fibroblasts through dysregulation of the TGF-β signalling pathway may be pivotal to this aberrant tissue repair and remodelling^5^. Lung fibroblasts are the principal cells in controlling ECM homeostasis by producing ECM proteins, matrix metalloproteases and tissue inhibitors of matrix metalloproteases. Transforming growth factor-β (TGF-β) is the main inducer of ECM production in lung fibroblasts. TGF-β levels are increased in COPD^5–8^ and alterations in the TGF-β signalling pathway have been demonstrated in COPD compared to control fibroblasts^9^.

MicroRNAs (miRNAs) regulate the vast majority of cellular processes and pathways by negatively influencing protein expression of multiple target genes^10^. These small non-coding RNAs play a role in various human diseases^11^. Several studies suggested that miRNAs contribute to COPD pathogenesis based on differential expression in e.g. whole blood, bronchoalveolar lavage cell fraction and lung tissue from COPD patients compared to smoking controls^12,13^. Previously, Ikari *et al*. compared miRNA expression changes between COPD and control fibroblasts (n = 5 per group)^14^ and selected miR-503, which was decreased in COPD fibroblasts, for further validation^15^. MiR-503 was demonstrated to enhance the production of VEGF, a direct target of miR-503^15^. Apart from these findings on miRNA expression in lung fibroblasts, limited information on differential miRNA expression in COPD fibroblasts is available. In our previous study, we identified 29 TGF-β-regulated miRNAs in primary parenchymal lung fibroblasts from control subjects^16^. Of these miRNAs, miR-455-3p and miR-21-3p were shown to target genes that are involved in the TGF-β and WNT signalling pathways, indicating a role of these miRNAs in tissue repair^16^. We hypothesize that TGF-β-regulated changes in miRNA expression in lung fibroblasts are involved in the pathogenesis of COPD.

To better understand the alterations in the TGF-β-regulated tissue repair response in COPD, it is of interest to identify miRNAs that are regulated by TGF-β in COPD and control lung fibroblasts and to identify miRNAs that are differentially regulated by TGF-β in COPD compared to control fibroblasts. In the present study, we used small RNA sequencing analysis; (1) to identify TGF-β-regulated miRNAs in clinically well-characterized severe COPD and control fibroblasts, (2) to identify which miRNAs are differentially regulated by TGF-β between COPD and control fibroblasts and (3) to determine miRNAs differentially expressed between COPD and control fibroblasts.

## Results

### Subject characteristics

Clinical characteristics of the 30 lung fibroblast and 35 lung tissue donors are shown in Table 1. Six of the lung fibroblasts donors in the control group were current smokers. The other 24 lung fibroblasts donors and all lung tissue donors were ex-smokers. No differences were identified in age and number of pack-years between controls and COPD patients, and also not between lung fibroblast and lung tissue donors.

**Table 1:** Subject characteristics.

Characteristics	Lung fibroblast donors		Lung tissue donors	
	Controls	COPD patients	Controls	COPD patients
N	15	15	17	18
Male/Female, n	7/8	6/9	8/9	6/12
Age (range), years^a^	65 (50–68)	57 (52–59)	59 (53–70)	55 (52–60)
Ex-/ current smoker, n	9/6	15/0	17/0	18/0
FEV_1_, %predicted^a,b^	94.7 (89.4–98.1)	18.4 (15.0–22.3)	89.2 (85.0–99.0)	16.0 (13.8 -23.5)
FEV_1_/FVC, %^a,c^	75.8 (72.5–79.1)	24.9 (20.5–28.7)	75.0 (70.2–79.0)	27.8 (26.2–36.0)
Pack-years, n^a^	32.5 (23.3–48.3)	37.5 (30.0–43.3)	32.0 (10.0–43.0)	30.0 (20.4–36.3)

^a^Median (Interquartile range).

^b^FEV_1_, % predicted, percentage of Forced Expiratory Volume in one second of the predicted normal value for an individual of the same sex, age and height.

^c^FEV_1_/FVC, Forced Expiratory Volume in one second/Forced Vital Capacity ratio expressed in percentage, a measurement for obstruction in the lungs.

### Differentially expressed miRNAs in response to TGF-β stimulation

**Figure 1:**
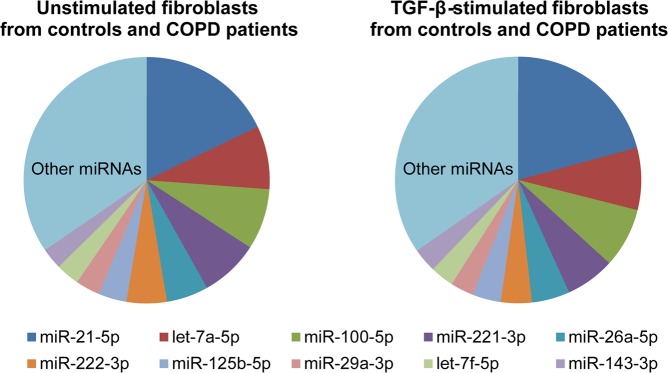
Top-10 most abundant miRNAs in lung fibroblasts of controls and COPD patients with/without TGF-β stimulation. Relative fractions of the read counts of the top-10 most abundant miRNAs in unstimulated (Left) and in TGF-β-stimulated lung fibroblasts (Right). The read counts in control and COPD lung fibroblasts were combined, as the expression levels of these ten miRNAs were similar in control and COPD fibroblasts.

As a control of successful TGF-β stimulation we first analysed the expression of *fibronectin-1* (*FN1*), *collagen type I alpha I* (*COL1A1*), and *alpha-smooth muscle actin* (*α-SMA)*, three known TGF-β-induced genes. A significant upregulation was observed for all three genes in both COPD and control fibroblasts (Supplementary Figure 1, p < 0.001). The total number of reads obtained of each sample and the percentages of reads mapping to miRBase Release 21 are shown in Supplementary Table 1. The ten most abundant miRNAs (Fig. 1) were expressed at similar levels in lung fibroblasts from control and COPD fibroblasts, either with or without TGF-β stimulation. The majority of the reads in all four groups were derived from miR-21-5p. Together, the top-10 most abundant miRNAs accounted for approximately 65% of all reads in unstimulated and TGF-β-stimulated lung fibroblasts (Fig. 1).

**Figure 2:**
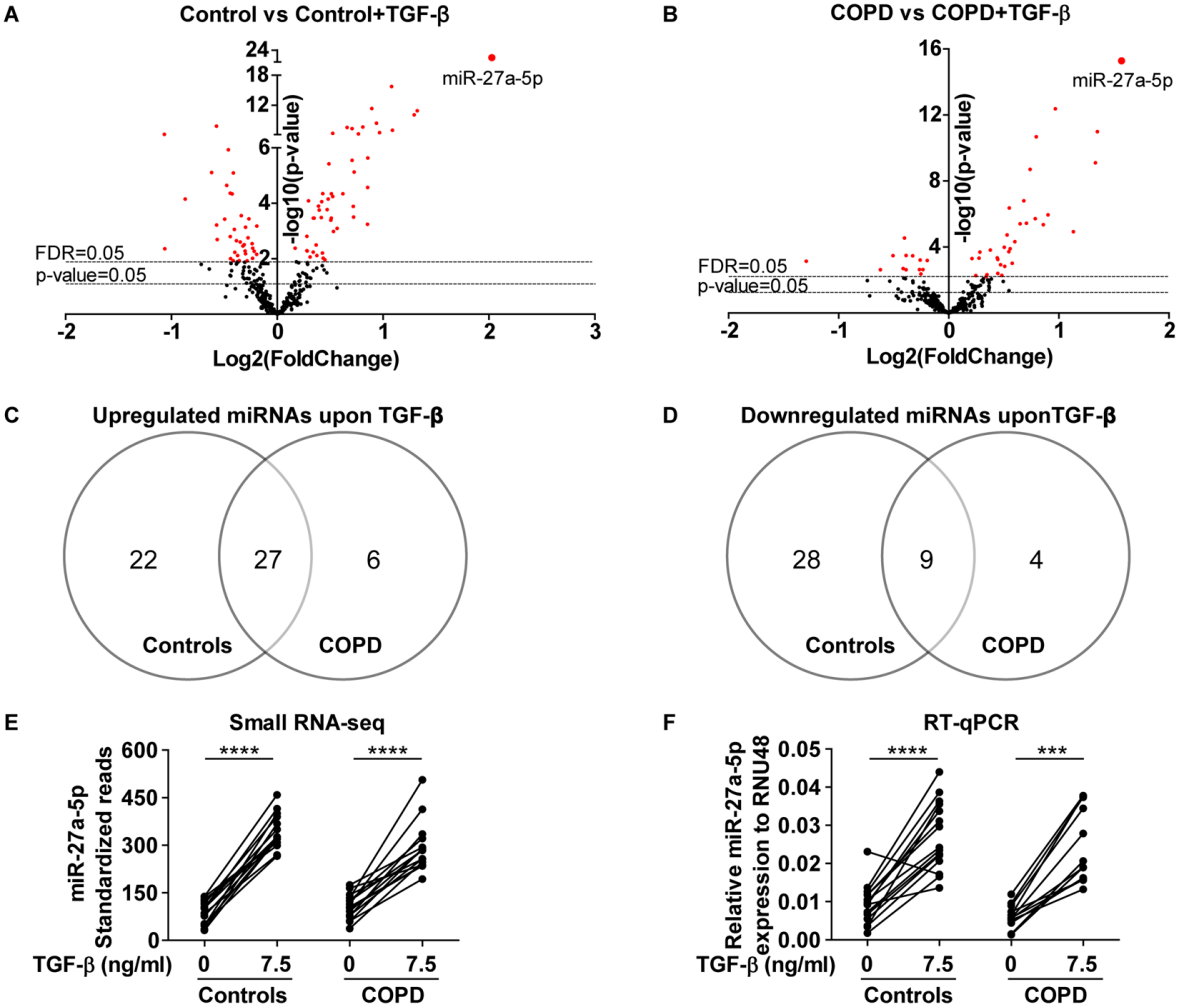
Differentially expressed miRNAs upon TGF-β stimulation in lung fibroblasts. (**A**,**B**) Volcano plot of the 349 miRNAs included in the analyses of the small RNA sequencing data. The lowest horizontal line represents the nominal p-value cut-off of 0.05. The upper horizontal line represents the FDR of 0.05. Differentially expressed miRNAs are indicated with red dots (FDR < 0.05). (**C**) Overlap of the upregulated miRNAs and (**D**) downregulated miRNAs after TGF-β stimulation in lung fibroblasts from controls and COPD patients. (**E**) MiR-27a-5p expression in the lung fibroblasts from controls and COPD patients with/without TGF-β stimulation based on the small RNA sequencing data. ****FDR < 0.0001. (**F**) Validation of miR-27a-5p expression in lung fibroblasts from 15 controls and 12 COPD patients with and without TGF-β stimulation using RT-qPCR. The data are presented as relative expression to RNU48 (2^−ΔCp^). ***p-value < 0.001, ****p-value < 0.0001.

Next, we identified miRNAs that were differentially expressed upon TGF-β stimulation in lung fibroblasts from controls and COPD patients. In control subjects, we identified 86 differentially expressed miRNAs (49 up- and 37 downregulated, FDR < 0.05, Fig. 2A, Supplementary Table 2). In COPD patients, 46 miRNAs were differentially expressed upon TGF-β stimulation (33 up- and 13 downregulated, FDR < 0.05, Fig. 2B, Supplementary Table 2). The overlap between the control and COPD fibroblasts was 27 for the TGF-β-induced and 9 for the TGF-β-repressed miRNAs (Fig. 2C,D). In total, 96 miRNAs were significantly differentially expressed upon TGF-β stimulation in lung fibroblasts of controls and/or COPD patients, including the most abundantly expressed miRNAs miR-21-5p, miR-26a-5p, miR-221-3p and miR-222-3p (Supplementary Table 2). The most significantly differentially expressed miRNA for both groups was miR-27a-5p with a fold change of 4.1 in control and 3.0 in COPD fibroblasts (Fig. 2E). The TGF-β-induced differential expression of miR-27a-5p was validated in the same lung fibroblasts using RT-qPCR (p-value < 0.001, Fig. 2F).

### MiRNAs differentially regulated by TGF-β in COPD compared to control fibroblasts

To identify miRNAs that are differentially regulated by TGF-β in COPD compared to control fibroblasts, we performed an interaction analysis on the 96 TGF-β-regulated miRNAs identified above. We found twelve miRNAs that responded differentially to TGF-β stimulation in lung fibroblasts from COPD compared to control fibroblasts at a nominal p-value < 0.05 (Table 2). For seven of them, the change in expression induced by TGF-β was detected only in control fibroblasts while there was no change in COPD fibroblasts. For two miRNAs, an increased expression was observed in COPD upon TGF-β treatment, while there was no change in control fibroblasts. Three miRNAs showed a change in the same direction, but in different degrees.

**Table 2:** MiRNAs differentially regulated by TGF-β in COPD compared to control fibroblasts.

MiRNA	p-value	FDR adjusted p-value	Change after TGF-β in	
			Control	COPD
miR-148b-3p	2.57E-03	0.18	−1.3*	1.0
miR-589-5p	5.35E-03	0.18	−1.3*	1.1
miR-376b-3p	5.71E-03	0.18	1.8*	1.1
miR-501-3p	1.39E-02	0.31	−1.4*	1.0
miR-99b-5p	1.67E-02	0.31	1.0	1.2*
miR-27a-5p	2.78E-02	0.31	4.1*	3.0*
miR-25-3p	3.18E-02	0.31	−1.3*	1.0
miR-370-5p	3.67E-02	0.31	1.1	1.5*
miR-136-3p	4.14E-02	0.31	1.3*	1.1
miR-21-3p	4.31E-02	0.31	2.1*	1.7*
let-7e-3p	4.33E-02	0.31	1.3*	1.1
miR-181a-2-3p	4.34E-02	0.31	1.6*	2.0*

^*^Significant TGF-β effect.

**Figure 3:**
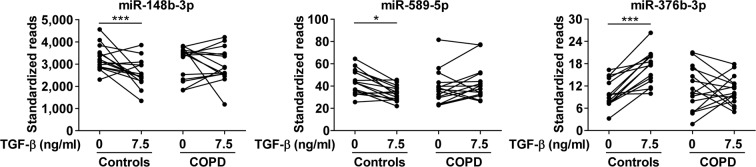
TGF-β ~ COPD interaction. Lung fibroblasts of COPD patients responded differently to TGF-β stimulation compared to those of controls with respect to expression of miR-148b-3p, miR-589-5p and miR-376b-3p (p-value miR-148b-3p = 0.0026, p-value miR-589-5p = 0.0053, p-value miR-376b-3p = 0.0057; FDR of interaction analysis all <0.25), based on small RNA sequencing data. *FDR TGF-β effect in controls <0.05, ***FDR TGF-β effect in controls <0.001.

After correcting for multiple testing, three miRNAs, miR-148b-3p, miR-589-5p and miR-376b-3p, remained significant at an FDR < 0.25 (Fig. 3). MiR-148b-3p and miR-589-5p levels were significantly decreased upon TGF-β stimulation in control fibroblasts, whereas no effect was observed in COPD fibroblasts. The levels of miR-376b-3p were significantly increased upon TGF-β stimulation in control fibroblasts, while again no change was observed in COPD fibroblasts. Despite reasonable read counts for miR-148b-3p, RT-qPCR revealed very high Cp values (34–37), precluding a reliable validation (data not shown). MiR-589-5p and miR-376b-3p had low read counts and were therefore not selected for RT-qPCR validation.

### Small RNA sequencing analysis of COPD and control lung fibroblasts

**Figure 4:**
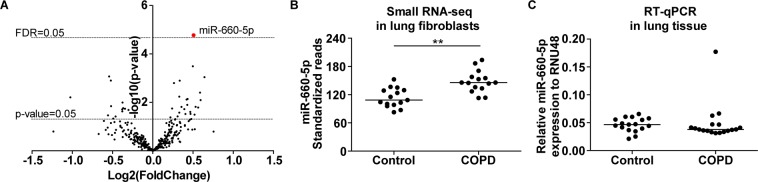
MiR-660-5p in lung fibroblasts and lung tissue from COPD patients. (**A**) Volcano plot of the 349 miRNAs included in the analyses of the small RNA sequencing data. The lowest horizontal line represents the nominal p-value cut-off of 0.05. The upper horizontal line represents the FDR of 0.05. MiR-660-5p, indicated with a red dot, was higher expressed in lung fibroblasts from COPD compared to controls (FC = 1.4, FDR = 5.9 × 10^−3^). (**B**) MiR-660-5p expression in the lung fibroblasts from controls and COPD patients based on the small RNA sequencing data. **FDR = 5.9 × 10^−3^. (**C**) MiR-660-5p expression in lung tissue from controls and COPD patients using RT-qPCR. The data are presented as relative expression to RNU48 (2^−ΔCp^).

Linear regression analysis of the 349 miRNAs obtained after filtering revealed one miRNA with a higher expression in lung fibroblasts from COPD patients at an FDR cut-off <0.05 (miR-660-5p, FC = 1.4, FDR p-value = 5.9 × 10^−3^, Fig. 4A,B). Additionally, 37 other miRNAs showed differential expression between COPD and control fibroblasts at a nominal p-value < 0.05 (Supplementary Table 3). Validation of the differential expression of miR-660-5p by RT-qPCR in lung fibroblasts was not feasible due to low expression levels (Cp values 33–37, data not shown). In the lung tissue samples used for replication, miR-660-5p expression levels were within the range of detection (Cp values 30–32), however, no differences were observed between lung tissue from COPD patients and from controls (Fig. 4C).

### Predicted targets of miR-27a-5p, miR-148b-3p and miR-660-5p

**Figure 5:**
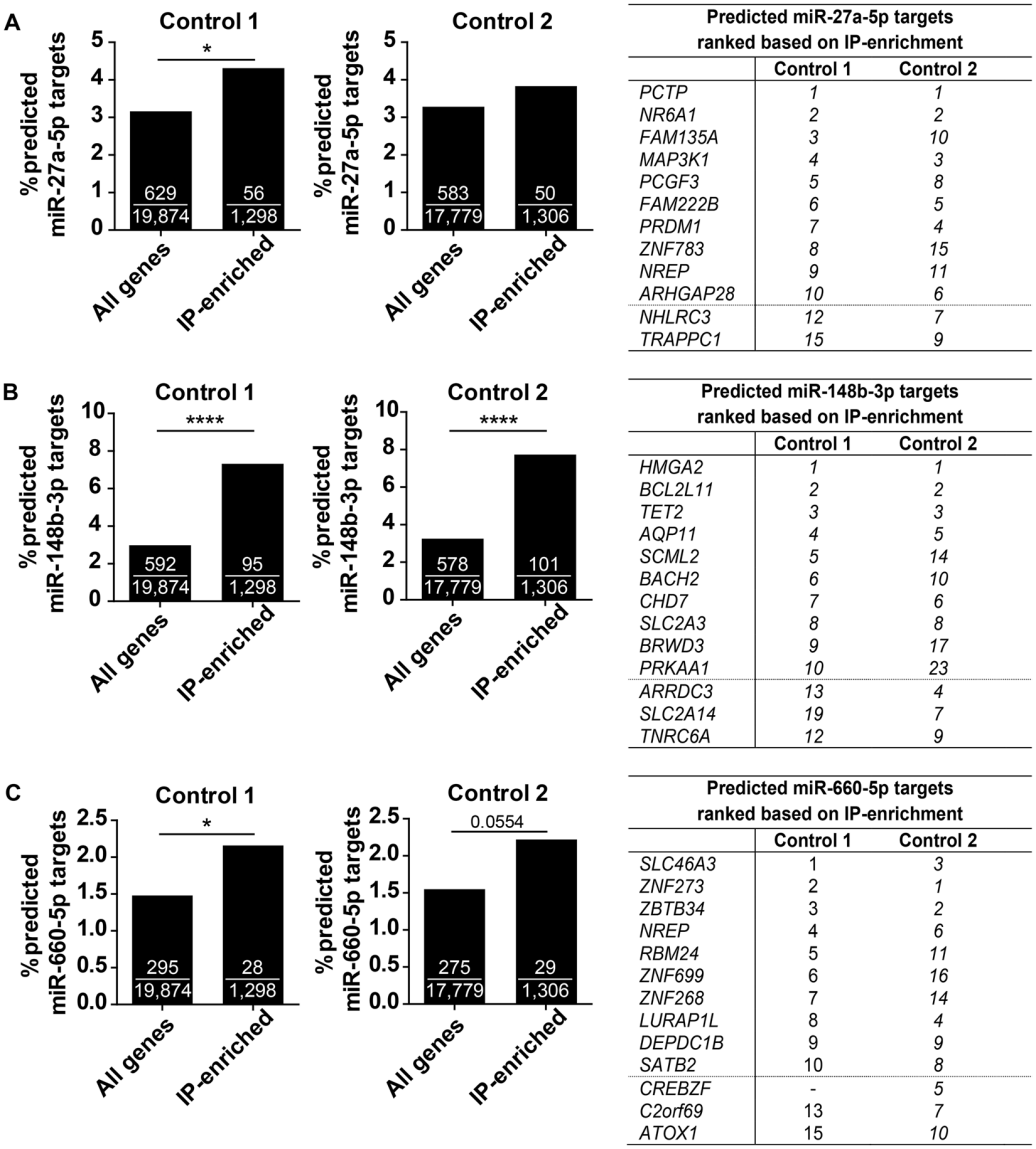
Ago2-IP-enrichment of predicted targets of miR-27a-5p, miR-148b-3p and miR-660-5p. The percentages of predicted targets of (**A**) miR-27a-5p, (**B**) miR-148b-3p and (**C**) miR-660-5p were calculated in all expressed genes and in the top-1,500 most IP-enriched probes in lung fibroblasts of two control subjects (Control 1 and 2). For miR-27a-5p, miR-148b-3p and miR-660-5p, 922, 677 and 421 predicted targets, respectively, were included in the analyses. Chi-square test was used to determine whether the number of predicted targets in the top-1,500 most Ago2-IP-enriched probes was significantly different from the expected based on the number of predicted targets in all expressed genes. All the other numbers in the bars are the number of predicted targets of each miRNA. *p-value < 0.05, ****p-value < 0.0001. The top-10 most IP-enriched predicted target genes are shown in the table. The numbers in the table indicate the rankings of the predicted target genes based on the IP-enrichment, i.e. 1 = most IP-enriched predicted target gene, 2 = second most IP-enriched target gene.

For the identification of target genes of miR-27a-5p, miR-148b-3p and miR-660-5p relevant in lung fibroblasts we re-analysed our previously published miRNA-targetome dataset (generated with Ago2-IP) of primary lung fibroblasts from two control subjects^16^. For miR-27a-5p a significant enrichment of miRNA target genes in the top-1,500 of most IP-enriched genes compared to all expressed genes was observed in one of the two controls (Fig. 5A). For miR-148b-3p, we observed a significant enrichment of predicted targets in both controls (p-value < 0.0001, Fig. 5B). For miR-660-5p, a significant (p-value < 0.05) and a borderline significant (p = 0.0554) enrichment of predicted target genes was observed (Fig. 5C).

The top-10 most IP-enriched predicted target genes for each of these three miRNAs are shown in Fig. 5, including the ranking in the IP-enrichment for each control. *Phosphatidylcholine Transfer Protein* (*PCTP*) was the most IP-enriched predicted target gene of the TGF-β-induced miR-27a-5p. Of the most enriched predicted target genes of miR-27a-5p, *Nuclear Receptor Subfamily 6 Group A Member 1* (*NR6A1*) and *PR/SET Domain 1* (*PRDM1*) are both involved in negative regulation of transcription, and *NHL Repeat Containing 3* (*NHLRC3*) and *Trafficking Protein Particle Complex 1* (*TRAPPC1*) are engaged in neutrophil mediated immunity (Supplementary Table 4). *High Mobility Group AT-Hook 2* (*HMGA2*) was the most IP-enriched predicted target of miR-148b-3p. Of the top-10 most enriched predicted miR-148b-3p target genes, seven genes play a role in the regulation of gene transcription. For miR-660-5p, *Solute Carrier Family 46 Member 3* (*SLC46A3*) and *Zinc Finger Protein 273* (*ZNF273*) were the most enriched predicted target genes. Five of the top-10 most IP-enriched predicted miR-660-5p target genes were involved in regulation of gene transcription.

## Discussion

Using small RNA sequencing, we identified 96 TGF-β-regulated miRNAs in lung fibroblasts from controls and/or COPD patients, from which miR-27a-5p was the most strongly TGF-β-induced miRNA. In addition, we identified miR-148b-3p, miR-589-5p and miR-376b-3p as differentially regulated by TGF-β in COPD compared to control fibroblasts. Of these miRNAs, only miR-148b-3p was highly expressed based on our small RNA sequencing data. Furthermore, we demonstrated higher expression of miR-660-5p in COPD compared to control fibroblasts. Using our previously published miRNA-targetome dataset, we identified multiple IP-enriched and predicted targets of miR-27a-5p, miR-148b-3p and miR-660-5p, representing possible targets through which these miRNAs may affect lung fibroblast function in COPD.

TGF-β stimulation had a strong effect on the miRNA expression profile in lung fibroblasts from COPD patients and controls. Interestingly, we found much less TGF-β-regulated miRNAs in COPD fibroblasts compared to controls. A previous study reported that the TGF-β release by COPD fibroblasts was elevated, whereas their repair responses were reduced compared to control fibroblasts^5^. This may suggest that COPD lung fibroblasts are also less responsive to TGF-β stimulation in our study. Of the TGF-β-regulated miRNAs, miR-27a-5p was the most prominent in lung fibroblasts from both COPD patients and controls. These findings were consistent with our previous study investigating TGF-β effects on control lung fibroblasts^16^. Interestingly, the interaction analysis suggested that the induction of miR-27a-5p upon TGF-β stimulation was lower in COPD compared to control fibroblasts, supporting the notion that COPD fibroblasts are less responsive to TGF-β than control fibroblasts. The most IP-enriched predicted target gene of miR-27a-5p was *PCTP*. This gene encodes a protein that regulates intermembrane transfer of phosphatidylcholine, which is one of the most abundant phospholipids in plasma membranes^17^. Our enrichment and pathway analysis suggest that miR-27a-5p is involved in negative regulation of transcription by targeting *NR6A1* and *PRDM1*. NR6A1 can repress gene expression by binding to DNA and recruiting DNA methyltransferases^18^. PRDM1 is a transcription factor that can repress β-interferon expression^19^. In addition, PRDM1 downregulates *p53* transcription by binding to its promoter, while p53 positively regulates transcription of *PRDM1*^20^. Knockdown of PRDM1 in human fibroblasts resulted in growth arrest^20^. Together this indicates that TGF-β-induced miR-27a-5p expression in lung fibroblasts may represent a mechanism to control TGF-β-induced cell proliferation via targeting *PRDM1*. Pathway analyses suggested that miR-27a-5p may also be involved in neutrophil mediated immunity by targeting *NHLRC3* and *TRAPPC1*. However, expression of these genes is not restricted to neutrophils; our previously published Ago2-IP dataset demonstrated high expression levels of these genes in primary lung fibroblasts^16^. A recent study showed that miR-27a-5p affected NF-κB signalling in human aortic endothelial cells^21^, but none of those NF-κB signalling genes were enriched in the miRNA-targetome of our lung fibroblasts.

In the interaction analyses, we found only miR-148b-3p with high read counts that responded differently at an FDR cut-off of 0.25 to TGF-β in lung fibroblasts from COPD patients compared to controls. MiR-148b-3p expression was lower upon TGF-β treatment in the controls and did not change in COPD patients. It is tempting to speculate that processes are activated in lung fibroblasts from controls in the presence of TGF-β, which may contribute to tissue repair and remodelling. This response may be attenuated in lung fibroblasts from COPD patients. In bronchial smooth muscle cells from asthma patients, miR-148b-3p was upregulated^22^. *HMGA2* was the most IP-enriched target gene of miR-148b-3p and was shown to be required for TGF-β-induced transcription of several genes, including *GATA6*^23^. It was suggested that *GATA6* may mediate the TGF-β-induced upregulation of *alpha-smooth muscle actin* (*α-SMA*) in fibroblasts from idiopathic pulmonary fibrosis with histopathology of usual interstitial pneumonia^24^. As miR-148b-3p was downregulated upon TGF-β-stimulation in lung fibroblasts from controls, it is plausible that miR-148b-3p may indirectly contribute to the TGF-β-induced upregulation of *α-SMA*. HMGA2 can also induce apoptosis and growth arrest in in human lung fibroblasts^25^, which may be due to the decreased miR-148b-3p expression after TGF-β treatment.

Of the other miRNAs identified in the interaction analysis, miR-99b-5p, miR-181a-2-3p, and miR-136-3p, have already been linked to COPD previously^26–28^. In addition, miR-25-3p and miR-181a-2-3p were found to be downregulated in COPD lung fibroblasts compared to controls in our current study (p-value < 0.05). For miR-21-3p, we previously showed regulation by TGF-β in control and COPD lung fibroblasts consistent with our current findings^16^.

We found a significantly higher expression of miR-660-5p in lung fibroblasts from COPD patients compared to controls. MiR-660-5p did not pop up in the previous miRNA profiling study of Ikari *et al*.^14^. Previously, circulating miR-660-5p levels were shown to be positively associated with FEV_1_/FVC of asthmatic children^29^. This positive association of miR-660-5p with lung function was not found in lung fibroblasts of severe COPD patients included in our study. A possible explanation for this might be that this miRNA has cell type and/or organ specific functions. Pathway analysis demonstrated that five predicted miR-660-5p targets, including the top gene *ZNF273*, are involved in regulating gene transcription. ZNF273 is a C_2_H_2_ zinc-finger motifs and Krüppel-associated box (KRAB)-domain-containing protein. Members of the KRAB-containing protein family are involved in transcriptional repression of RNA polymerase promoters, binding and splicing of RNA, and control of nucleolus function^30^. In addition, another target, *SLC46A3* has been shown to be upregulated in bronchial epithelial cells of COPD compared to controls^31^. This is different from our findings of higher miR-660-5p levels in COPD lung fibroblasts, which would rather be associated with lower *SLC46A3* expression in COPD. Again, this might be due to different involvement in cell type specific functions. Previously, miR-660-5p was shown to directly target *MDM2*^32^, which was among the IP-enriched target gene in one of our controls^16^. MDM2 is involved in degradation of p53 protein^33^ and downregulation of p53 in control lung fibroblasts increased the proliferation rate and the migration and invasion capacity^33^. A miR-660-5p-regulated decrease in MDM2 expression in lung fibroblasts may thus modulate fibroblast proliferation and motility via p53. Interestingly, because in line with the increased miR-660-5p in COPD, it was reported that p53 protein levels were elevated in emphysematous lung tissue^34,35^.

Whereas small RNA sequencing revealed differential regulation upon TGF-β stimulation for miR-148b-3p in COPD fibroblasts and differential expression of miR-660-5p, both miRNAs could not be validated using RT-qPCR in the same samples, since the expression levels were too low to be quantified reliably. Especially for miR-148b-3p which had high read counts in the small RNA sequencing data and very strong enrichment of predicted target genes, this was a surprise. It may be explained by lack of specificity of the assays. Although overall read counts of miR-660-5p were relatively low, differences in small RNA sequencing data were clear and significant enrichment of predicted target genes in lung fibroblast does suggest that this miRNA is active in these cells. Further investigation, including biological validation, is needed to confirm our results.

In conclusion, we showed that TGF-β affects expression of a considerable number of miRNAs in primary lung fibroblasts. Three of these TGF-β-regulated miRNAs responded differently to TGF-β in COPD compared to control fibroblasts. In contrast, only one miRNA was differentially expressed in COPD fibroblasts. Altered miRNA regulation, i.e. differential response to TGF-β stimulation in COPD compared to control fibroblasts and differentially expressed miR-660-5p in COPD, may be one of the mechanisms underlying aberrant tissue repair and remodelling in COPD.

## Materials and Methods

### Subjects

**Figure 6:**
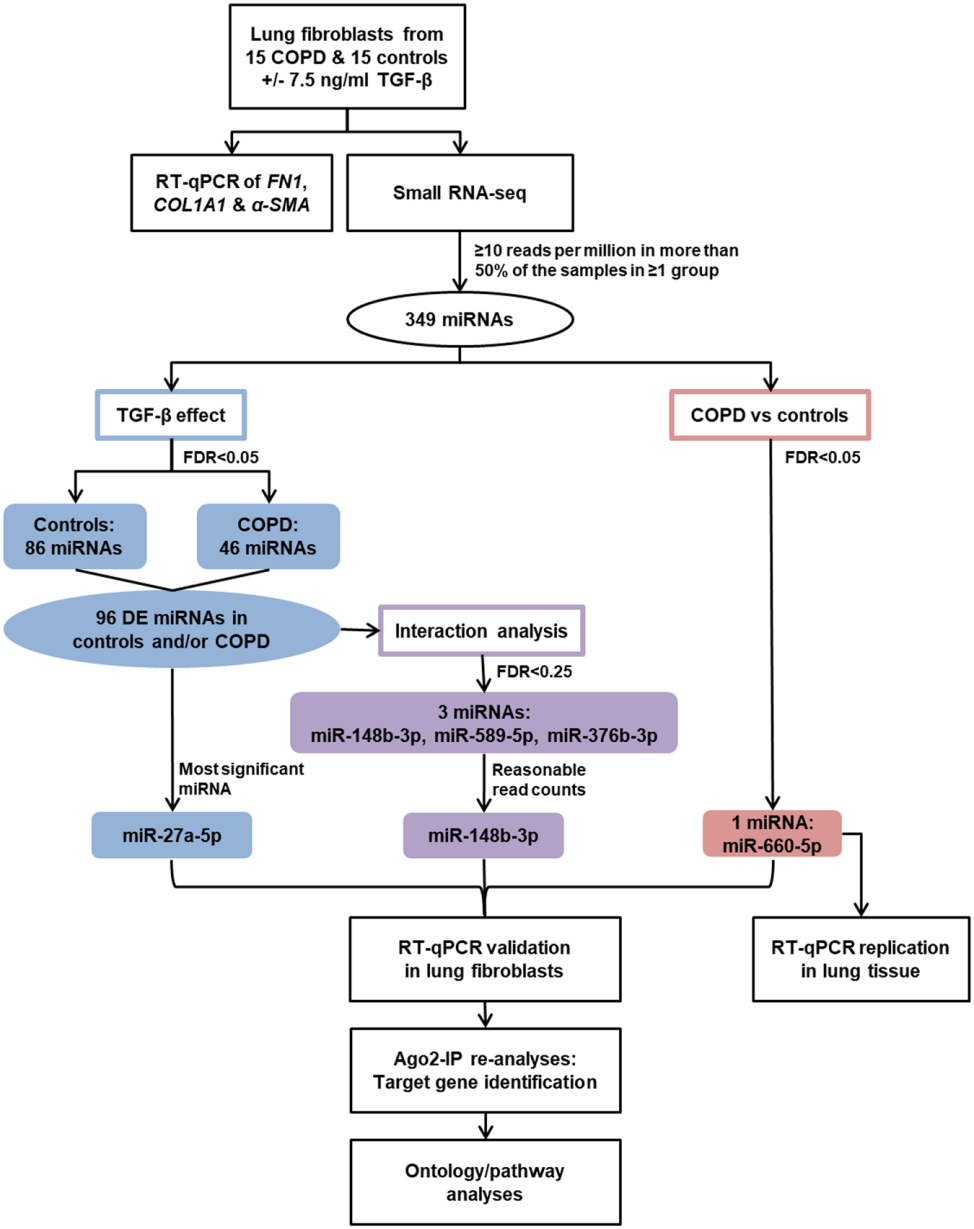
Workflow FDR = false discovery rate, DE = differentially expressed, Ago2-IP = argonaute 2-immunoprecipitation.

The workflow of this study is shown in Fig. 6. Primary fibroblasts were obtained from 15 stage IV COPD patients undergoing lung transplant surgery and 15 non-COPD controls with normal lung function undergoing lung tumour resection surgery. For the latter group lung fibroblasts were isolated from left-over parenchymal lung tissue located far away from the tumour without histological abnormalities^36,37^. Lung tissue from 18 stage IV COPD patients and 17 non-COPD controls was used to replicate the results.

This study was performed in accordance with the national ethical and professional guidelines on the use human body material (“Code of conduct; Dutch federation of biomedical scientific societies”; https://www.federa.org/codes-conduct) and the Research Code of the University Medical Centre Groningen (https://www.umcg.nl/SiteCollectionDocuments/English/Researchcode/umcg-research-code-2018-en.pdf). The left-over lung material, which was used to isolate lung fibroblasts or to replicate the results, was at the time of the experiments not subject to the act on medical research involving human subjects in the Netherlands. Therefore, an ethics waiver was provided by the Medical Ethical Committee of the University Medical Centre Groningen (METc UMCG). All samples and clinical information were de-identified before experiments were performed.

### Cell culture, TGF-β stimulation and RNA isolation

Primary fibroblasts were isolated from lung parenchyma using the explant technique as described previously^38^. The fibroblasts were cultured in complete medium consisting of Ham’s F12 medium, 10% (v/v) fetal calf serum (FCS), 100 U/ml penicillin/streptomycin and 200 mM L-glutamine (all from Lonza, Breda, The Netherlands). The experiments were performed on fibroblasts at passage 5. Lung fibroblasts were stimulated with 7.5 ng/ml TGF-β1 (R&D Systems, Abingdon, UK) in complete Ham’s F12 medium containing 0.5% (v/v) FCS for 24 h. Total RNA was isolated from primary parenchymal lung fibroblasts and lung tissues using TRIzol (Invitrogen, Carlsbad, CA, USA), according to the protocol of the manufacturer. The RNA concentration was measured with a NanoDrop 1000 Spectrophotometer (Thermo Scientific, Wilmington, DE, USA).

### cDNA synthesis and qPCR for ECM genes and α-SMA

To confirm effective TGF-β stimulation, expression levels of several known TGF-β-regulated genes *FN1*, *COL1A1* and *α-SMA* in lung fibroblasts^39,40^ were determined by RT-qPCR, as described previously^16^. *18S rRNA* (*18S*) and *RNA polymerase II* (*RP2*) were used as reference genes. Positive and negative controls were included in each run. The formula 2^−ΔCp^ was used to calculate the relative mRNA expression levels.

### Small RNA sequencing

Libraries were generated with the NEXTflex Small RNA-seq kit V3 (Bioo Scientific, Uden, The Netherlands) using total RNA (approximately 1 μg) of the lung fibroblasts with/without TGF-β stimulation and sequenced on the NextSeq 500 (Illumina, San Diego, CA, USA) according to the protocol of the manufacturer. TrimGAlore 0.3.7 was used to trim the adapter sequences of the raw reads. Subsequently, the reads were allocated to the human miRNAs (miRBase Release 21, http://www.mirbase.org/) allowing one mismatch using the miRDeep2 V2.0.0.8 software^41^. The reads of miRNAs with the same mature sequence were summed up. Only miRNAs with ≥10 reads per million in more than half of the samples in at least one group (lung fibroblasts of COPD patients or controls, with or without TGF-β stimulation) were included. After applying these filtering steps, 349 miRNAs were left for further analyses.

### cDNA syntheses and qPCR for miRNAs

To validate and replicate the small RNA sequencing results, RT-qPCR was performed as described previously^16,42^. Briefly, cDNA was synthesized using 10 ng total RNA of the parenchymal fibroblasts and/or lung tissues and reverse transcription primers from TaqMan® microRNA Assay kits, small nucleolar RNA, C/D box 48 (RNU48) (Assay ID: 001006), hsa-miR-27a* (Assay ID: 002445), hsa-miR-148b (Assay ID: 000471) and efu-miR-660 (Assay ID: 475114_mat) (Applied Biosystems, Carlsbad, CA, USA). Assays were selected based on the most abundant miRNA sequence identified in the small RNA sequencing data. Next, qPCR was performed in LightCycler 480 Probes Master reaction mix (Roche Diagnostics GmbH, Mannheim, Germany) and the above mentioned TaqMan microRNA assays. RNU48 was used as reference gene, and positive and negative controls were included in each run. The relative miRNA expression levels were calculated using the formula 2^−ΔCp^.

### Ago2-IP and gene ontology/pathway analyses

Data of our previously published argonaute-2-immunoprecipitation (Ago2-IP) experiments in primary lung fibroblasts from two control subjects were used to identify genes targeted by miRNAs selected in this study, as described previously^16^. Control 1 had in total 19,874 expressed genes of which the top-1,500 IP-enriched probes consisted of 1,298 unique genes^16^. Control 2 had in total 17,779 expressed genes of which the top-1,500 IP-enriched probes encounter for 1,306 unique genes^16^. For poorly conserved miRNAs, predicted targets with a cumulative weighted context++ score ≤0.2 were included in the analyses (TargetScan version 7.2^43^). For broadly conserved miRNAs, predicted targets with conserved binding sites were considered. To assess the potential functional relevance of individual miRNAs, we identified the top-10 most IP-enriched and TargetScan predicted genes. For each miRNA, the top-10 most enriched predicted target genes were subjected to Enrichr^44,45^ to identify the biological processes (gene-set library: GO_Biological_Process_2018) and Reactome pathways (gene-set library: Reactome_2016). We focused on pathways for which at least two of the top-10 enriched genes are involved in, without taking the significance of the process/pathway enrichment into account.

### Statistical analyses

The Mann Whitney U test was used to compare differences in subject characteristics between COPD patients and control subjects, and between the fibroblast and lung tissue donors (IBM SPSS Statistics version 22). Small RNA sequencing data were analysed in R (v3.5.2)^46^ with the Bioconductor-Limma package (v3.38.3)^47^.

Three separate analyses were conducted; (1) a linear regression model (TGF-β - baseline) was performed to investigate the effect of TGF-β overall on miRNA expression in controls and COPD fibroblasts, separately, (2) interaction analysis ((COPD TGF-β - COPD baseline) - (control TGF-β - control baseline)) was conducted to investigate whether miRNAs are differentially regulated by TGF-β in COPD compared to control fibroblasts, and (3) a linear regression model (COPD baseline - control baseline) was run to look at the differences of miRNA expression between COPD and control fibroblasts at baseline. For the interaction analyses, we focused on miRNAs that were differentially expressed upon TGF-β stimulation in fibroblasts from COPD patients and/or controls. These models were adjusted for age, gender, smoking status and library preparation batch. Results were corrected for multiple testing using the Benjamini-Hochberg false discovery rate (FDR).

The TGF-β effect on miRNA expression levels determined by RT-qPCR was analysed using the one-sided paired Wilcoxon signed rank test. Enrichment of predicted target genes in the Ago2-IP was assessed by a chi-square test on the percentage of predicted targets in the top-1,500 enriched probes compared to the percentage of predicted targets in all expressed genes. A p-value below 0.05 was considered statistically significant.

## Supplementary information

Supplementary info

## Data Availability

The small RNA sequencing dataset generated for this manuscript is available for collaboration upon request.
