# Supplementary material for: Marked TGF-β-regulated miRNA expression changes in both COPD and control lung fibroblasts
Source: Sci Rep. 2019 Dec 3;9:18214. doi: 10.1038/s41598-019-54728-4 (PMC6890791; doi:10.1038/s41598-019-54728-4)
Supplement: Supplementary file 1 — Supplementary info [file 41598_2019_54728_MOESM1_ESM.pdf]

Marked TGF-β-regulated miRNA expression changes in both COPD and control lung fibroblasts

J Ong[1,2], A Faiz[2,3,4], W Timens[1,2], M van den Berge[2,3], MM Terpstra[5], K Kok[5], A van den Berg[1], J Kluiver[1,#], CA Brandsma[1,2,#,*]

[1] University of Groningen, University Medical Centre Groningen, Department of Pathology and Medical Biology, Groningen, The Netherlands.

[2] University of Groningen, University Medical Centre Groningen, Groningen Research Institute for Asthma and COPD (GRIAC), Groningen, The Netherlands.

[3] University of Groningen, University Medical Centre Groningen, Department of Pulmonary Diseases, Groningen, The Netherlands.

[4] University of Technology Sydney, Respiratory Bioinformatics and Molecular Biology (RBMB) Faculty of Science, Ultimo NSW 2007, Australia

[5] University of Groningen, University Medical Centre Groningen, Department of Genetics, Groningen, The Netherlands

[#] Co-last authors

* Corresponding author

E-mail: c.a.brandsma@umcg.nl (CAB)

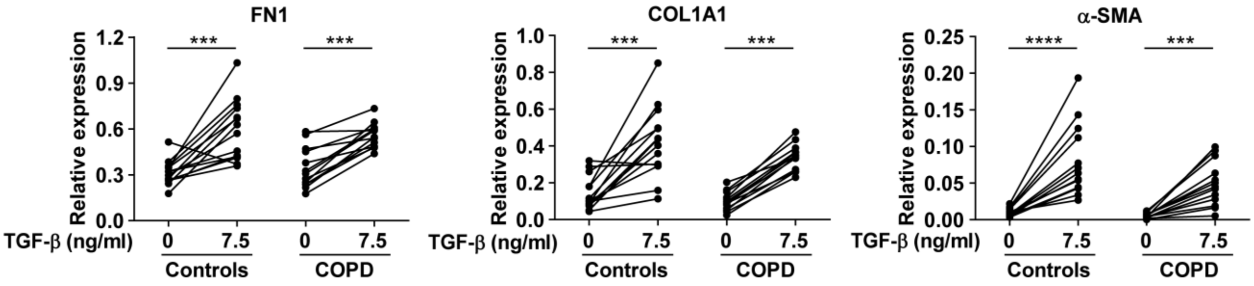

Supplementary Figure 1. Upregulation of ECM genes and α-SMA upon TGF-β stimulation in primary parenchymal lung fibroblasts. Effective TGF-β stimulation in lung fibroblasts was confirmed by the TGF-β-induced *FN1* (*fibronectin 1*), *COL1A1* (*collagen type I alpha I*) and *α-SMA* (*alpha smooth muscle actin*). *18S rRNA* (*18S*) and *RNA polymerase II* (*RP2*) were used as reference genes. The relative mRNA expression level was calculated using the formula $2^{-\Delta Cp}$.The gene expression of one control and two COPD patients are missing due to technical reasons. ***p-value<0.001, ****p-value<0.0001.

Supplementary Table 1. Number of reads obtained by small RNA sequencing and percentage mapped to miRBase

		Total after trimming	Mapping to miRBase release 21	
Group	Sample	Reads	Reads	Percentage
Controls	Control 1	8,613,228	954,721	11.1%
	Control 2	5,967,036	1,764,687	29.6%
	Control 3	13,582,970	1,310,675	9.6%
	Control 4	13,664,253	1,194,114	8.7%
	Control 5	13,176,488	2,280,947	17.3%
	Control 6	5,447,263	1,256,637	23.1%
	Control 7	15,633,258	1,583,379	10.1%
	Control 8	7,078,531	2,219,801	31.4%
	Control 9	5,551,348	2,396,604	43.2%
	Control 10	8,892,472	1,461,870	16.4%
	Control 11	16,834,054	1,048,153	6.2%
	Control 12	7,789,705	1,213,615	15.6%
	Control 13	12,844,518	1,314,038	10.2%
	Control 14	11,511,590	1,999,201	17.4%
	Control 15	9,459,738	1,080,041	11.4%
Controls+TGF-β	Control 1	17,197,731	1,521,799	8.8%
	Control 2	13,613,880	3,003,560	22.1%
	Control 3	12,674,248	1,849,882	14.6%
	Control 4	11,621,975	2,639,920	22.7%
	Control 5	10,226,650	1,950,132	19.1%
	Control 6	7,046,034	999,718	14.2%
	Control 7	25,079,361	3,113,395	12.4%
	Control 8	14,211,350	1,166,374	8.2%
	Control 9	9,570,145	1,577,600	16.5%
	Control 10	9,483,992	667,585	7.0%
	Control 11	11,639,209	997,702	8.6%
	Control 12	14,850,821	1,705,361	11.5%
	Control 13	6,963,231	2,701,890	38.8%
	Control 14	12,850,125	1,259,353	9.8%
	Control 15	12,519,447	2,406,067	19.2%

Supplementary Table 1. Continued

		Total after trimming	Mapping to miRBase release 21	
**Group**	**Sample**	**Reads**	**Reads**	**Percentage**
COPD	COPD 1	3,798,325	1,248,672	32.9%
	COPD 2	13,795,142	1,236,137	9.0%
	COPD 3	4,950,861	1,587,487	32.1%
	COPD 4	14,425,189	1,898,930	13.2%
	COPD 5	13,161,948	2,378,268	18.1%
	COPD 6	13,443,281	1,189,760	8.9%
	COPD 7	7,891,188	1,851,893	23.5%
	COPD 8	15,810,780	1,314,752	8.3%
	COPD 9	12,340,571	892,651	7.2%
	COPD 10	4,371,237	1,381,489	31.6%
	COPD 11	12,712,033	1,434,376	11.3%
	COPD 12	26,010,757	3,077,254	11.8%
	COPD 13	11,634,021	932,389	8.0%
	COPD 14	11,815,943	1,581,333	13.4%
	COPD 15	17,981,408	3,119,150	17.3%
COPD+TGF-β	COPD 1	11,939,405	2,376,454	19.9%
	COPD 2	8,367,109	3,142,737	37.6%
	COPD 3	11,233,282	1,916,242	17.1%
	COPD 4	12,375,463	2,273,604	18.4%
	COPD 5	11,167,125	1,857,121	16.6%
	COPD 6	11,926,975	1,057,745	8.9%
	COPD 7	9,597,946	2,128,859	22.2%
	COPD 8	6,286,481	2,091,980	33.3%
	COPD 9	10,554,139	998,231	9.5%
	COPD 10	14,033,738	1,096,912	7.8%
	COPD 11	8,904,112	1,405,006	15.8%
	COPD 12	19,065,046	2,044,723	10.7%
	COPD 13	6,331,380	1,482,085	23.4%
	COPD 14	14,171,053	2,637,211	18.6%
	COPD 15	15,439,873	1,250,475	8.1%

Supplementary Table 2. Differentially expressed miRNAs upon TGF-β stimulation in lung fibroblasts from controls and COPD patients

miRNA	Controls			COPD		
	FC	p-value	FDR	FC	p-value	FDR
miR-27a-5p	4.1	5.45E-21	1.90E-18	3.0	5.20E-16	1.81E-13
miR-503-5p	2.5	1.37E-11	1.20E-09	2.5	1.03E-11	1.20E-09
miR-23a-5p	2.5	9.00E-11	6.28E-09	1.9	1.11E-06	4.32E-05
miR-424-3p	2.1	1.10E-07	3.49E-06	2.5	7.75E-10	5.41E-08
miR-21-3p	2.1	1.86E-16	3.24E-14	1.7	2.09E-11	1.82E-09
miR-424-5p	2.0	3.23E-07	9.39E-06	1.8	4.45E-06	1.20E-04
miR-154-3p	1.9	4.07E-09	2.37E-07	1.5	1.92E-04	3.19E-03
miR-27b-5p	1.9	4.86E-12	5.65E-10	1.7	1.98E-09	1.15E-07
miR-455-5p	1.8	2.34E-06	4.80E-05	1.4	5.20E-03	4.13E-02
miR-376b-3p	1.8	2.70E-05	3.92E-04			
miR-181b-3p	1.8	5.73E-04	4.17E-03	2.2	1.17E-05	2.92E-04
miR-125a-3p	1.8	2.45E-08	1.07E-06	1.6	3.87E-06	1.13E-04
miR-214-5p	1.7	5.71E-07	1.42E-05	1.4	1.17E-03	1.20E-02
miR-23b-5p	1.7	7.49E-06	1.29E-04	1.7	1.90E-06	6.63E-05
miR-100-3p	1.6	3.18E-04	2.74E-03			
miR-4521	1.6	1.31E-04	1.27E-03			
miR-181a-3p	1.6	5.18E-08	1.81E-06	1.6	1.57E-07	7.82E-06
miR-455-3p	1.6	2.84E-06	5.51E-05	1.6	3.57E-06	1.13E-04
miR-181a-2-3p	1.6	2.89E-08	1.12E-06	2.0	4.20E-13	7.33E-11
miR-132-5p	1.5	4.59E-05	5.54E-04			
miR-4455	1.5	8.07E-04	5.41E-03			
miR-1185-1-3p	1.4	1.04E-03	6.72E-03			
let-7f-1-3p	1.4	5.73E-05	6.66E-04	1.4	5.63E-04	6.77E-03
miR-125b-1-3p	1.4	4.55E-07	1.22E-05	1.3	1.53E-04	2.66E-03
miR-410-3p	1.4	4.56E-05	5.54E-04			
miR-143-5p	1.4	3.46E-04	2.74E-03	1.5	1.28E-04	2.35E-03
miR-487a-3p	1.4	4.06E-04	3.02E-03	1.5	4.76E-05	9.78E-04
miR-145-3p	1.4	3.77E-06	6.93E-05	1.5	4.30E-07	1.87E-05
miR-495-3p	1.4	7.08E-05	7.72E-04			
miR-132-3p	1.4	1.70E-04	1.60E-03	1.4	4.48E-04	6.01E-03
miR-210-5p	1.4	1.10E-02	4.63E-02			
let-7a-2-3p	1.4	9.56E-03	4.17E-02			
let-7e-3p	1.3	4.53E-05	5.54E-04			
miR-92a-1-5p	1.3	6.23E-03	2.94E-02	1.4	1.48E-03	1.48E-02
miR-24-2-5p	1.3	8.85E-05	9.09E-04			
miR-199a-5p	1.3	3.27E-04	2.74E-03			
miR-136-3p	1.3	1.76E-04	1.62E-03			
miR-376a-3p	1.3	1.27E-04	1.27E-03			
miR-431-5p	1.3	7.69E-03	3.44E-02			
miR-214-3p	1.3	3.18E-03	1.76E-02	1.4	1.02E-04	1.98E-03
miR-199-3p	1.3	3.44E-04	2.74E-03			
miR-376c-3p	1.3	3.46E-04	2.74E-03			
miR-4286	1.3	5.83E-03	2.83E-02	1.3	6.24E-03	4.73E-02
miR-1260b	1.2	8.71E-03	3.85E-02			
miR-21-5p	1.2	8.14E-05	8.61E-04			
miR-337-3p	1.2	5.27E-03	2.63E-02			
miR-22-5p	1.2	5.15E-03	2.63E-02			
miR-27a-3p	1.2	1.55E-03	9.84E-03	1.2	5.40E-03	4.19E-02
miR-23a-3p	1.1	4.19E-03	2.22E-02	1.2	4.98E-04	6.21E-03
miR-26a-5p	-1.1	6.66E-04	4.65E-03			

Supplementary Table 2. Continued

miRNA	Controls			COPD		
	FC	p-value	FDR	FC	p-value	FDR
let-7g-5p	-1.1	6.96E-03	3.20E-02			
miR-26b-5p	-1.2	5.43E-03	2.67E-02			
miR-128-3p	-1.2	4.18E-03	2.22E-02			
miR-140-5p	-1.2	9.69E-03	4.18E-02			
miR-361-3p	-1.2	2.86E-03	1.64E-02			
miR-454-3p	-1.2	3.65E-04	2.83E-03	-1.2	6.06E-04	7.05E-03
miR-30d-5p	-1.2	1.76E-03	1.08E-02			
miR-500a-3p	-1.2	1.20E-02	4.87E-02			
miR-1301-3p	-1.2	6.63E-03	3.08E-02			
miR-221-3p	-1.2	7.29E-04	4.99E-03	-1.2	4.28E-03	3.55E-02
miR-1307-3p	-1.2	5.25E-03	2.63E-02			
miR-7-5p	-1.2	3.11E-03	1.75E-02			
miR-25-3p	-1.3	3.48E-03	1.90E-02			
miR-362-5p	-1.3	2.78E-04	2.49E-03			
miR-10a-5p	-1.3	2.51E-03	1.46E-02			
miR-28-3p	-1.3	1.18E-02	4.84E-02			
miR-29b-3p	-1.3	7.67E-03	3.44E-02			
miR-589-5p	-1.3	2.26E-03	1.34E-02			
miR-1287-5p	-1.3	5.92E-03	2.83E-02			
miR-532-5p	-1.3	8.83E-04	5.82E-03			
miR-92b-3p	-1.3	8.13E-06	1.29E-04			
miR-146b-3p	-1.3	1.17E-02	4.84E-02			
miR-148b-3p	-1.3	4.60E-05	5.54E-04			
miR-2277-5p	-1.4	9.99E-03	4.25E-02			
miR-30c-5p	-1.4	4.34E-05	5.54E-04	-1.3	3.24E-04	4.70E-03
miR-501-3p	-1.4	1.59E-03	9.92E-03			
miR-222-3p	-1.4	1.18E-06	2.56E-05	-1.3	3.37E-04	4.70E-03
miR-155-5p	-1.4	2.27E-05	3.45E-04			
miR-500a-5p	-1.4	3.73E-04	2.83E-03	-1.4	3.34E-04	4.70E-03
miR-584-5p	-1.5	2.03E-03	1.22E-02			
miR-30a-3p	-1.5	6.12E-04	4.36E-03			
miR-221-5p	-1.5	1.59E-08	7.95E-07	-1.3	2.86E-05	6.23E-04
miR-218-5p	-1.5	7.74E-06	1.29E-04	-1.3	2.01E-03	1.94E-02
miR-1303	-1.8	7.08E-05	7.72E-04			
miR-4485-3p	-2.1	4.39E-03	2.28E-02	-2.5	7.19E-04	7.85E-03
miR-222-5p	-2.1	7.40E-07	1.72E-05	-1.5	2.38E-03	2.07E-02
miR-370-5p				1.5	9.52E-04	1.01E-02
miR-320b				1.4	1.84E-05	4.29E-04
miR-25-5p				1.4	3.96E-03	3.37E-02
miR-99b-3p				1.3	4.77E-03	3.87E-02
miR-181b-5p				1.2	2.04E-04	3.23E-03
miR-99b-5p				1.2	4.65E-04	6.01E-03
miR-103a-3p				-1.1	6.53E-04	7.35E-03
miR-660-5p				-1.2	2.32E-03	2.07E-02
miR-130b-3p				-1.2	2.32E-03	2.07E-02
miR-331-5p				-1.3	2.36E-03	2.07E-02

Supplementary Table 3. Differentially expressed miRNAs between COPD and controls (nominal p-value<0.05)

miRNA	FC	p-value	FDR
miR-660-5p	1.4	1.69E-05	5.90E-03
miR-362-5p	1.4	3.25E-04	5.67E-02
miR-155-5p	-1.5	8.55E-04	7.43E-02
miR-137	1.6	9.18E-04	7.43E-02
miR-181a-2-3p	-1.4	1.26E-03	7.43E-02
miR-23b-3p	1.3	1.28E-03	7.43E-02
miR-103a-3p	1.2	1.60E-03	7.98E-02
miR-500a-5p	1.5	3.98E-03	1.73E-01
miR-331-3p	1.3	5.81E-03	2.17E-01
miR-598-3p	-2.0	6.24E-03	2.17E-01
miR-409-5p	1.4	7.26E-03	2.17E-01
miR-502-3p	1.3	7.67E-03	2.17E-01
miR-532-3p	1.5	8.08E-03	2.17E-01
miR-106b-3p	-1.3	1.02E-02	2.54E-01
miR-495-3p	1.3	1.27E-02	2.86E-01
miR-28-3p	-1.4	1.44E-02	2.86E-01
miR-98-3p	1.4	1.54E-02	2.86E-01
miR-22-5p	1.3	1.54E-02	2.86E-01
miR-369-5p	1.5	1.65E-02	2.86E-01
miR-7-5p	-1.3	1.75E-02	2.86E-01
miR-27b-3p	1.2	1.79E-02	2.86E-01
miR-145-3p	-1.3	1.85E-02	2.86E-01
miR-500a-3p	1.3	1.91E-02	2.86E-01
miR-25-3p	-1.3	1.96E-02	2.86E-01
miR-92a-3p	-1.2	2.06E-02	2.88E-01
miR-299-3p	-1.3	2.38E-02	3.19E-01
miR-154-3p	1.4	2.48E-02	3.20E-01
miR-337-3p	1.3	2.65E-02	3.31E-01
miR-656-3p	1.4	3.02E-02	3.52E-01
miR-452-5p	1.3	3.03E-02	3.52E-01
miR-501-5p	1.4	3.22E-02	3.63E-01
miR-335-5p	-1.5	3.45E-02	3.76E-01
miR-655-3p	1.2	3.81E-02	3.96E-01
miR-25-5p	-1.4	3.86E-02	3.96E-01
miR-107	1.2	4.17E-02	4.16E-01
miR-374a-3p	-1.4	4.69E-02	4.55E-01
miR-4455	1.4	4.89E-02	4.55E-01
miR-24-3p	1.2	4.96E-02	4.55E-01

Supplementary Table 4. Biological processes and pathways of selected IP-enriched predicted targets of miR-27a-5p, miR-148b-3p and miR-660-5p

Biological process	miR-27a-5p	miR-148b-3p	miR-660-5p
Regulation of gene expression (GO:0010468)		PRKAA1;CHD7;HMGA2;BACH2	ZNF699;ZNF268
Regulation of transcription, DNA-templated (GO:0006355)		CHD7;HMGA2;BACH2;BRWD3	CREBZF;SATB2;ZNF699;ZNF268
Regulation of transcription from RNA polymerase II promoter (GO:0006357)	NR6A1;PRDM1	TET2;HMGA2;BRWD3	SATB2;ZNF268
Regulation of nucleic acid-templated transcription (GO:1903506)		CHD7;HMGA2;BACH2	ZNF699;ZNF268
Positive regulation of gene expression (GO:0010628)		PRKAA1;HMGA2	
Positive regulation of transcription, DNA-templated (GO:0045893)		TET2;HMGA2	
Positive regulation of transcription from RNA polymerase II promoter (GO:0045944)		TET2;HMGA2	
Negative regulation of transcription, DNA-templated (GO:0045892)	NR6A1;PRDM1		CREBZF;ZNF268
Negative regulation of transcription from RNA polymerase II promoter (GO:0000122)	NR6A1;PRDM1		
Neutrophil mediated immunity (GO:0002446)	TRAPPC1;NHLRC3		
Neutrophil activation involved in immune response (GO:0002283)	TRAPPC1;NHLRC3		
Neutrophil degranulation (GO:0043312)	TRAPPC1;NHLRC3		
Regulation of apoptotic process (GO:0042981)		PRKAA1;BCL2L11;HMGA2	
Positive regulation of apoptotic process (GO:0043065)		BCL2L11;HMGA2	
Positive regulation of programmed cell death (GO:0043068)		BCL2L11;HMGA2	
Negative regulation of apoptotic process (GO:0043066)		PRKAA1;HMGA2	
Negative regulation of programmed cell death (GO:0043069)		PRKAA1;HMGA2	
Regulation of cellular macromolecule biosynthetic process (GO:2000112)		CHD7;HMGA2;BACH2	ZNF699;ZNF268
Positive regulation of macromolecule metabolic process (GO:0010604)		PRKAA1;HMGA2	
**Reactome Pathways**	**miR-27a-5p**	**miR-148b-3p**	**miR-660-5p**
Gene Expression_Homo sapiens_R-HSA-74160	NR6A1;PRDM1	PRKAA1;TET2;TNRC6A	ZNF273;ZNF699;ZNF268
Generic Transcription Pathway_Homo sapiens_R-HSA-212436	NR6A1;PRDM1	PRKAA1;TNRC6A	ZNF273;ZNF699;ZNF268
Transcriptional Regulation by TP53_Homo sapiens_R-HSA-3700989		PRKAA1;TNRC6A	
TP53 Regulates Metabolic Genes_Homo sapiens_R-HSA-5628897		PRKAA1;TNRC6A	
Cellular Senescence_Homo sapiens_R-HSA-2559583		HMGA2;TNRC6A	
Cellular responses to stress_Homo sapiens_R-HSA-2262752		PRKAA1;HMGA2;TNRC6A	
Signal Transduction_Homo sapiens_R-HSA-162582		PRKAA1;BCL2L11;TNRC6A	
Signalling by NGF_Homo sapiens_R-HSA-166520		BCL2L11;TNRC6A	
Transmembrane transport of small molecules_Homo sapiens_R-HSA-382551		AQP11;SLC2A3	
